# The effects of different fatigue levels on brain–behavior relationships in driving

**DOI:** 10.1002/brb3.1379

**Published:** 2019-09-30

**Authors:** Kuan‐Chih Huang, Chun‐Hsiang Chuang, Yu‐kai Wang, Chi‐Yuan Hsieh, Jung‐Tai King, Chin‐Teng Lin

**Affiliations:** ^1^ Department of Electrical and Computer Engineering National Chiao Tung University Hsinchu Taiwan; ^2^ Brain Research Center National Chiao Tung University Hsinchu Taiwan; ^3^ Department of Computer Science and Engineering National Taiwan Ocean University Keelung Taiwan; ^4^ CIBCI, Centre for Artificial Intelligence, FEIT University of Technology Sydney Sydney NSW Australia

**Keywords:** brain dynamics, electroencephalograms, fatigue, longitudinal assessment

## Abstract

**Background:**

In the past decade, fatigue has been regarded as one of the main factors impairing task performance and increasing behavioral lapses during driving, even leading to fatal car crashes. Although previous studies have explored the impact of acute fatigue through electroencephalography (EEG) signals, it is still unclear how different fatigue levels affect brain–behavior relationships.

**Methods:**

A longitudinal study was performed to investigate the brain dynamics and behavioral changes in individuals under different fatigue levels by a sustained attention task. This study used questionnaires in combination with actigraphy, a noninvasive means of monitoring human physiological activity cycles, to conduct longitudinal assessment and tracking of the objective and subjective fatigue levels of recruited participants. In this study, degrees of effectiveness score (fatigue rating) are divided into three levels (normal, reduced, and high risk) by the SAFTE fatigue model.

**Results:**

Results showed that those objective and subjective indicators were negatively correlated to behavioral performance. In addition, increased response times were accompanied by increased alpha and theta power in most brain regions, especially the posterior regions. In particular, the theta and alpha power dramatically increased in the high‐fatigue (high‐risk) group. Additionally, the alpha power of the occipital regions showed an inverted U‐shaped change.

**Conclusion:**

Our results help to explain the inconsistent findings among existing studies, which considered the effects of only acute fatigue on driving performance while ignoring different levels of resident fatigue, and potentially lead to practical and precise biomathematical models to better predict the performance of human operators.

## Graphical abstract

In this manuscript, we performed a longitudinal experiment over 20 weeks to investigate the brain dynamics and behavioral changes of individuals under different levels of fatigue during a sustained attention task. Our results help to explain the inconsistent findings among existing studies, which considered the effects of only acute fatigue on driving performance while ignoring different resident fatigue levels.

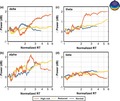

## 1 INTRODUCTION

Fatigue behind the wheel is assumed to be a crucial factor in the failure of drivers to avoid automobile crashes, which can lead to accidents, injuries, and fatalities (Fairclough & Graham, 1999; Hanowski, Wierwille, & Dingus, 2003; Sexton, Thomas, & Helmreich, 2000). Especially during long‐term, monotonous, or nighttime driving, (acute) fatigue (or drowsiness) frequently occurs, reducing drivers' performance. Hence, a comprehensive understanding of drowsy driving is an urgent necessity to enable researchers to develop drowsiness countermeasures for real‐life applications. Many imaging biomarkers relevant to drowsiness, such as eye closure, eye blinking (Caffier, Erdmann, & Ullsperger, 2003), and head nodding (Ji, Zhu, & Lan, 2004), have been used to monitor the cognitive state of drivers. However, false alarms are likely to occur, since these facial attributes are not always accompanied by drowsiness (Horne & Reyner, 1999).

In the past few decades, several studies have reported that drowsiness‐related behavioral lapses are accompanied by spectral changes in electroencephalograms (EEGs; Davidson, Jones, & Peiris, 2007; Huang et al., 2016; Kecklund & Akerstedt, 1993; Lin et al., 2010; Makeig & Inlow, 1993; Makeig & Jung, 1995; Peiris, Jones, Davidson, & Bones, 2006). Thus, many EEG‐based drowsiness monitoring and detection technologies have recently been developed for driving applications (Huang et al., 2016; Lin, Huang, Chuang, Ko, & Jung, 2013; Wang et al., 2014). The previous literatures have shown that brain oscillations in the alpha (8–12 Hz) and theta (4–7 Hz) bands are associated with driving lapses or with fluctuations in task performance under drowsiness (Huang et al., 2016; Huang, Jung, Chuang, Ko, & Lin, 2012; Jung, Makeig, Stensmo, & Sejnowski, 1997; Lin et al., 2010; Lin, Nascimben, King, & Wang, 2018). Another researcher reported significant increases only in theta power, frequency of theta bursts, and length of EEG theta activity episodes between alert and poor/drowsy performance, during prolonged driving, or with progressive deterioration of drivers' vigilance levels (Seen, Tamrin, & Meng, 2010).

Additionally, analyzing the ratio of theta power to alpha power suggests that alpha activity gradually decreases and is replaced by increasing theta activity during microsleep episodes (Boyle, Tippin, Paul, & Rizzo, 2008; Daniel, 1967). However, alpha, (theta + alpha)/beta, and alpha/beta power were observed to trend upward as driving error increased (Campagne, Pebayle, & Muzet, 2004; Taniguchi & Takaoka, 2001) or as fatigue gradually occurred (Eoh, Chung, & Kim, 2005; Jap, Lal, Fischer, & Bekiaris, 2009; Lal & Craig, 2001; Schier, 2000; Simon et al., 2011). Furthermore, several studies (Glass & Riding, 1999; Ota, Toyoshima, & Yamauchi, 1996) have noted that alpha power follows a biphasic trend (an inverted U‐shaped curve) as behavioral performance (or arousal level) decreases in some situations. As mentioned above, EEG results, especially in the alpha band, varied across studies. One purpose of the present study is to find the crucial factor that results in these inconsistent findings.

Most previous studies were conducted within well‐controlled settings. For example, each participant was instructed to maintain an alcohol‐ and caffeine‐restricted diet for 1 day before each experiment and required to complete a questionnaire about his or her sleeping habits; all participants had normal work and rest schedules, got enough sleep, and had not stayed up late at any time in a period of several days before the experiment. However, in the real world, individual daily physiological states are likely to be less uniform. There is still no subjective measurement for long‐term tracking of participants' fatigue state on a daily basis. Hence, it remains unclear how to incorporate changing fatigue levels into a brain–behavior model for real‐world applications.

There is literature showing that varying levels of fatigue in humans can induce homeostatic changes in the brain (Shenoy, Krauledat, Blankertz, Rao, & Muller, 2006). Therefore, we hypothesize that varying levels of fatigue may confound the observed relationship between brain dynamics and behavioral performance, thus affecting drowsiness detection mechanisms. In this study, we aim to investigate the effect of different fatigue levels on the brain–behavior relationship in driving. A longitudinal study was performed using an integrated daily sampling system (DSS) to track the fatigue states of multiple participants; the data were acquired from subjective reports (questionnaires), such as the Karolinska Sleepiness Scale (KSS) and the Fatigue Visual Analog Scale (FVAS), and from actigraphy conducted daily over a 20‐week period. Actigraphy, which is integrated into the DSS alongside the questionnaires, can continuously monitor the rest/activity cycles of the subject to assess fatigue levels, which is expressed by an effectiveness score. According to the effectiveness scores from actigraphy, fatigue states were divided into three different levels (high, reduced, and normal risks). All participants were scheduled to conduct the sustained attention task on three occasions at each of three levels of fatigue in order to explore the effect of different fatigue levels on simulated driving performance and corresponding informative EEG features. Finally, we established brain–behavior models (i.e., the relationship between EEG dynamics and task performance) that take into account different levels of fatigue for drowsy driving applications.

## 2 MATERIALS AND METHODS

### 2.1 Subjects

Seventeen healthy subjects (13 males and four females) aged 22.4 ± 1.5 years were recruited to participate in this study. All subjects were right‐handed, had normal or corrected‐to‐normal vision, and were not taking any medications known to affect cognitive function. None of the subjects had a history of central or peripheral neurological impairments, brain injury, alcohol abuse, diabetes, or drug addiction. The Institutional Review Board of National Chiao Tung University, Taiwan, approved the study. All subjects were first given an orientation session describing the procedures for the experiment and their responsibilities during the long‐term study, and they were informed about the experimental materials, features, and processes and required to read and sign a consent form before the experiments.

### 2.2 Experimental equipment

#### 2.2.1 Actigraphy monitoring device

A Fatigue Science Readiband actigraph (Fatigue Science) was issued to each participant during the study. The Readiband is a wrist‐worn actigraphy device that objectively and automatically characterizes sleep timing, duration, and quality, as well as an estimated percentage effectiveness score based on the patented Sleep, Activity, Fatigue, & Task Effectiveness (SAFTE) model (Kaida et al., 2006). The SAFTE model has been validated in independent laboratory studies and operational human factors investigations (Hursh et al., 2004; Van Dongen, Baynard, Maislin, & Dinges, 2004). Effectiveness scores describe how cognitive effectiveness, reaction time, and fatigue risk are affected by sleep quality, sleep quantity, and sleep/wake timing. The model uses sleep data to calculate an effectiveness score.

The SAFTE model mathematically simulates the main physiological processes that determine the level of fatigue (i.e., deficiency in performance effectiveness) at any given point in time. It contains a circadian process that represents the way in which the body clock influences both performance and circadian regulation, as well as a sleep‐reservoir process that represents the way in which recovery sleep is affected by bedtime, wake time, sleep quality, sleep quantity, sleep debt, the circadian timing of sleep, and any type of sleep fragmentation (waking up during the night). The SAFTE model provides real‐time effectiveness scores and determines when fatigue levels will reach a point where safety and performance are at risk.

#### 2.2.2 Self‐reporting questionnaires

Self‐reporting questionnaires, including the Karolinska Sleepiness Scale (KSS) and the Fatigue Visual Analog Scale (FVAS), were implemented to enable subjects to record their psychometric responses to fatigue, sleep, and stress. The KSS has been used extensively to measure subjective sleepiness and was originally validated with ambulatory EEG (Akerstedt & Gillberg, 1990; Kecklund & Akerstedt, 1993). The KSS was administered to participants on a daily basis to record subjective sleepiness. Participants indicate which level best reflected the psychophysical state they had experienced in the last 10 min. The KSS is a ten‐point scale (1 = extremely alert; 3 = alert; 5 = neither alert nor sleepy; 7 = sleepy but no difficulty remaining awake; and 9 = very sleepy, great effort to keep awake, fighting sleep; Akerstedt & Gillberg, 1990).

The FVAS has proven to be a simple yet effective tool (Lee, Hicks, & Ninomurcia, 1991). It is a sliding scale from “not at all fatigued” to “extremely fatigued.” For this experiment, the participant responded by placing a cursor on a line, and the device translated the location of the cursor to a number from 0 to 100.

#### 2.2.3 Virtual reality scene

Virtual reality (VR)‐based monotonous highway driving experiments were performed in a driving simulator that mimicked realistic driving situations in a dark, sound‐reduced room. The VR scenes simulated driving at a constant speed (100 km/hr) on a four‐lane divided highway, with the car randomly drifting away from the center of the cruising lane to the left or right side with equal probability to simulate driving on nonideal road surfaces or with poor alignment. The road was straight and uniform. Moreover, there was no traffic or other stimuli appeared in the VR scene, simulating a driving situation that is likely to induce drowsiness. The scenes were updated at 60 frames per second.

#### 2.2.4 EEG acquirement

During the experiment, EEG activity was recorded by the SynAmps system (Compumedics Ltd.) using a 64‐channel scalp electrode array (Ag/AgCl electrodes) with a unipolar reference at the mastoid. The EEG electrodes were placed according to a modified international 10–20 system. Contact impedance between EEG electrodes and the cortex was calibrated to <10 kΩ. The EEG data were recorded with a 32‐bit quantization level at a sampling rate of 1,000 Hz and preprocessed with a 50‐Hz low‐pass filter and a 0.5‐Hz high‐pass filter.

### 2.3 Experimental paradigm

Each participant was provided a wrist‐worn actigraph and trained in its use and how to operate the system and log daily data. Beginning at the orientation session, the participants were required to wear the Readiband continuously during the entire study period (i.e., 20 weeks) in order to objectively and automatically monitor their daily sleep patterns, rest‐activity cycle, and fatigue. Within an hour after awakening each morning, subjects were instructed to complete the self‐reporting questionnaires, including the subjective measures of fatigue/sleepiness and stress. Additionally, the effectiveness score (ES, 0%–100%) displayed on the Readiband was registered manually. The ES, an actigraph‐based sleep/wake score, was estimated by a biomathematical model of alertness (Hursh et al., 2004) built into the Readiband. In this study, we defined the *normal* group as having a daily effectiveness score near the MEAN + standard deviation (*SD*; The MEAN and *SD* were calculated over approximately 1 month). Subjects with effectiveness scores lower than the MEAN−*SD* were considered the *high‐risk* group. Those with effectiveness scores lying between the normal and high‐risk ranges were categorized as the *reduced* group. Participants were asked to wear the actigraph continuously for the duration of the study.

The data from the Readiband were automatically uploaded to a server which was maintained by the researchers in this laboratory. All the participants received notifications (by text message) to report for experimental trials within 12 hr if their conditions fit the experimental requirements. If it was not possible for a given participant to come in for testing within that period, or if there was a scheduling conflict in the laboratory, he or she was re‐evaluated the following day to determine whether he or she was still classified in the same readiness category after another night. If not, monitoring continued until an appropriate or high‐risk, reduced, or normal state was reached again.

Because we wished to conduct the experimental sessions with participants under well‐rested, sleep‐deprived, and sleep‐restricted states as they naturally occur in the real world, we could not predetermine or counterbalance the times when participants were scheduled for testing because we could not control when they would experience those states. To accommodate this limitation inherent in observational research, we tested each participant in 2‐week windows in whatever state he or she happens to be in for the first three experimental sessions, and for the remainder of the experimental sessions, they were scheduled when their sleep patterns and subjective states were in the states yet to be tested.

**Figure 1:**
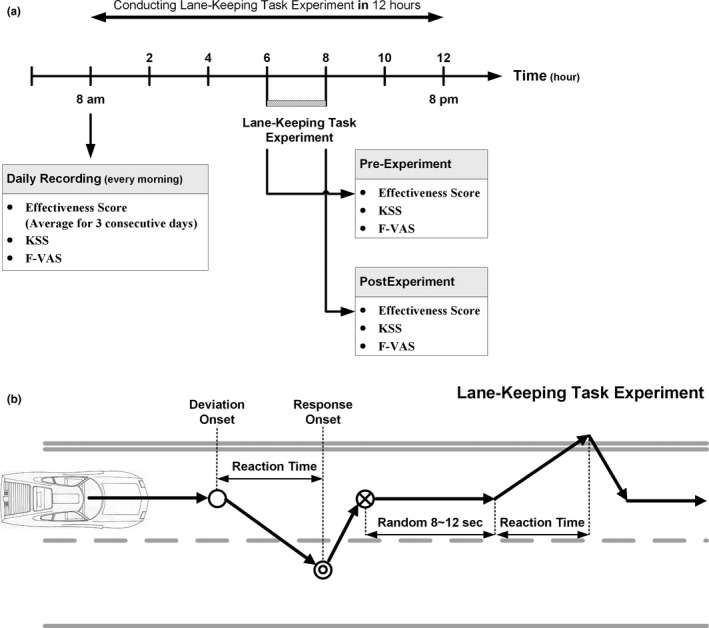
Experimental session paradigm. (a) The timeline of the experimental session. The KSS score, FVAS score, and ES were recorded at three time points. One point was in the morning, and the other two points were immediately before and after the experiment. Note that the experiment was conducted within 12 hr (usually within 8 hr) after the subject was notified by text message. (b) Event‐related lane‐keeping tasks. The solid black arrows represent the driving trajectory. The empty circle represents deviation onset. The double circle represents response onset. The circle with a cross represents the end of the response. The driver's RT is the time interval from deviation onset (empty circle) to response onset (double circle). The end of the response (circle with a cross) means that the driver has steered the car back into the original lane. The next deviation begins at 8–12 s after the end of the previous response (adapted from Huang, Jung, & Makeig, 2007)

Figure 1a shows the time sequence of the experiment session, from the morning measurement of fatigue to the end of the experiment. In this session, the participants conducted the sustained attention task experiment (Figure 1b). The program simulated driving a car at a certain speed (100 km/hr) on the highway at night, and the car automatically drifted away from the cruising lane to the left or right side with equal probability; participants were instructed to steer the vehicle back to the cruising lane as fast as possible after becoming aware of the deviation. If the participants did not respond to the lane‐perturbation event, for example, if they fell asleep, the vehicle could hit the left or right curb within 2.5 and 1.5 s, respectively.

The vehicle would then continue to move along the curb until it returned to the original lane. Each lane‐departure event was defined as a “trial” that included three critical moments: “Deviation onset” is the moment when the car starts to drift away, “response onset” represents the moment when the participant perceives the drift and begins to steer the car back to the cruising lane, and “response offset” is the moment when the car returns to the center of the cruising lane and the participant ceases to rotate the steering wheel. The next lane‐departure event occurred 8–12 s after the “response offset.” Reaction time was defined as the interval between deviation onset and response onset in a trial. In the interest of creating driving conditions likely to induce fatigue, there were no other vehicles or stimuli that might disturb the driver's attention. Participants' cognitive states and driving performance were monitored via a surveillance video camera and the vehicle trajectory throughout the experiment.

### 2.4 Data analysis

The recorded 62‐channel EEG signals were first inspected to remove bad EEG channels and then down‐sampled to 250 Hz. To observe the fluctuation in EEG signals at specific events, we extracted the continuous 62‐channel EEG signals into 9‐s epochs, time locked to 2 s before and 7 s after each deviation onset. The epochs contaminated by noise signals (muscle activity, blinking, eye movement, or environmental noise) were eliminated manually to minimize their influence on subsequent analysis.

Independent component analysis (ICA; Bell & Sejnowski, 1995, Makeig, Bell, Jung, & Sejnowski, 1996) was applied to decompose EEG signals into temporally independent time courses corresponding to brain and nonbrain sources using EEGLAB (Delorme & Makeig, 2004). The 62‐channel EEG signals were separated into 62 independent components, based on the assumption that EEG signals at the sensors were linear mixtures of activation of distinct brain and nonbrain sources whose time courses were statistically independent.

To identify comparable independent components across subjects, we grouped components from multiple subjects into component clusters based on their scalp maps, equivalent dipole locations, and baseline power spectra of component activations (Delorme & Makeig, 2004; Jung et al., 2001). The time courses of activation for the components of interest were selected and transferred into the frequency domain by the fast Fourier transform (FFT). The dynamic changes, defined as tonic changes in the EEG signals, were measured from the cruising period before the deviation onset in each epoch.

The average power spectra were then obtained by averaging across time points to obtain a mean baseline. For each channel in each session, the tonic power spectra of all epochs (trials) were sorted by their RTs and then normalized by subtracting the mean power spectra of the “alert trials” with the shortest RTs (lowest 10% of all RT‐sorted trials). Finally, to identify the trend of tonic power spectra in different levels of fatigue, we sorted all trials (epochs) by reaction time for each level of fatigue.

### 2.5 Statistical analysis with hierarchical linear modeling

In this study, longitudinal daily data (103–151 days) and experimental session data (6–9 experiments) were collected from 17 subjects over the course of a semester. We used these data to find the association between subjective questionnaires and objective sleep measurements. Such diary and session data recorded over prolonged periods, nested within participants and experimental test sessions, are naturally multilevel data. Therefore, a multilevel modeling approach was needed. Using *multilevel random coefficient modeling* (Nezlek, 2001; Woltman, Feldstain, MacKay, & Rocchi, 2012), we applied level 1 analyses to model the within‐subject variability of the data recorded repeatedly over extended time periods and level 2 analyses to model variability across subjects over time.

This approach has been used to illustrate the daily relationship between mood and sleep across 2 weeks (Mccrae et al., 2008). Multilevel analysis was conducted using mixed models in SPSS software to distinguish between‐ and within‐individual sources. Specifications for the multilevel models were selected following Peugh and Enders (2005) to determine the best‐fitting model for the variables in this study.

There are two levels in the random coefficient regression model. The level 1 model refers to the within‐person or individual change model (i.e., repeated measurements over time) and describes the longitudinal changes in each individual (i.e., the variation within the individual over time). The level 2 model estimates the average within‐person initial status and rate of change over a predictor variable.

## 3 RESULTS

### 3.1 Relationship among objective and subjective measures of fatigue

**Figure 2:**
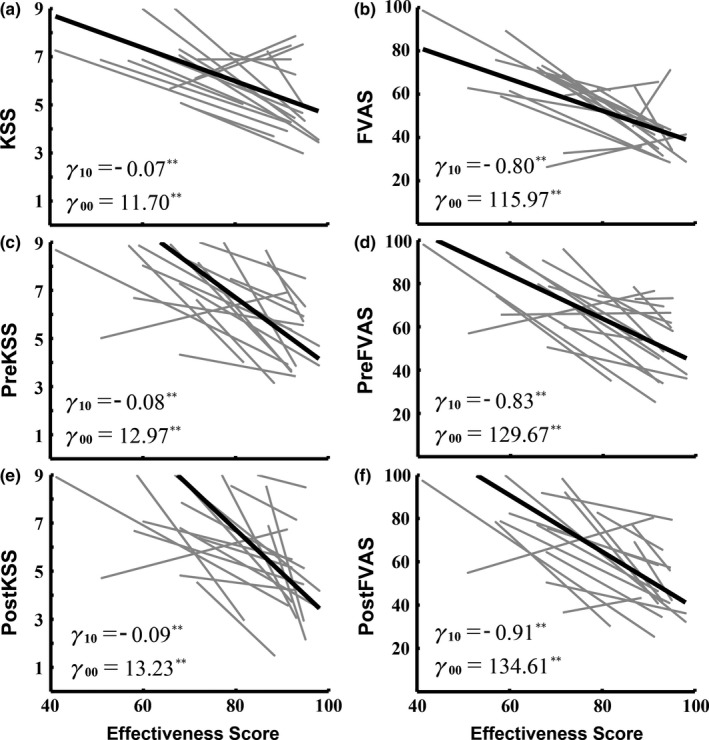
The estimating regression between subjective questionnaire scores (KSS & FVAS) and objective fatigue measurement (ES) at three time points in the day (morning, before the experiment, and after the experiment). The value on the *X*‐axis is the ES, and the values on the *Y*‐axis are the KSS and FVAS scores. ** *p*‐value <.01

Figure 2 shows the relations between objective sleep information measurement (*X*‐axis) and subjective questionnaires (*Y*‐axis; gray lines, individual regression; black lines, group mean) during different sessions. In each row of the figure, one graph shows the objective measurement (ES) and a subjective questionnaire (KSS or FVAS) in the morning on the day of the sustained attention task, another one (PreKSS or PreFVAS vs. ES) shows the values immediately before the sustained attention task, and the third reflects the measurements immediately after the sustained attention task (PostKSS or PostFVAS vs. ES). The whole dataset was collected from 17 subjects over the course of a semester (20 weeks) in this study. The coefficients *γ*_10_ and *γ*_00_ from the univariate mixed model regression used to predict the ES after experiment preparation represent the slope and intercept, respectively. The coefficient *γ*_10_ is the overall mean slope across subjects and sessions, and *γ*_00_ is the overall (grand) mean intercept across subjects and sessions.

The subfigures in the left column of Figure 2 show the significant linear decreases in KSS (*γ*_10_ = −0.07, standard error [*SE*] = 0.01, *p* < .01; *γ*_00_ = 11.70, *SE* = 0.86, *p* < .01; in Figure 2a), PreKSS (*γ*_10_ = −0.08, *SE* = 0.017, *p* < .01; *γ*_00_ = 12.97, *SE* = 1.398, *p* < .01; in Figure 2c), and PostKSS (*γ*_10_ = −0.09, *SE* = 0.022, *p* < .01; *γ*_00_ = 13.23, *SE* = 1.515, *p* < .01; in Figure 2e). The subfigures in the right column of Figure 2 show the significant linear decreases in FVAS (*γ*_10_ = −0.8, *SE* = 0.125, *p* < .01; *γ*_00_ = 115.97, *SE* = 10.477, *p* < .01; in Figure 2b), PreFVAS (*γ*_10_ = −0.83, *SE* = 0.136, *p* < .01; *γ*_00_ = 129.67, *SE* = 11.699, *p* < .01; in Figure 2d), and PostFVAS (*γ*_10_ = −0.91, *SE* = 0.166, *p* < .01; *γ*_00_ = 134.61, *SE* = 14.184, *p* < .01; in Figure 2f). These results show a clear correlation between subjective measurements (KSS and FVAS) and objective measurements (ES).

**Figure 3:**
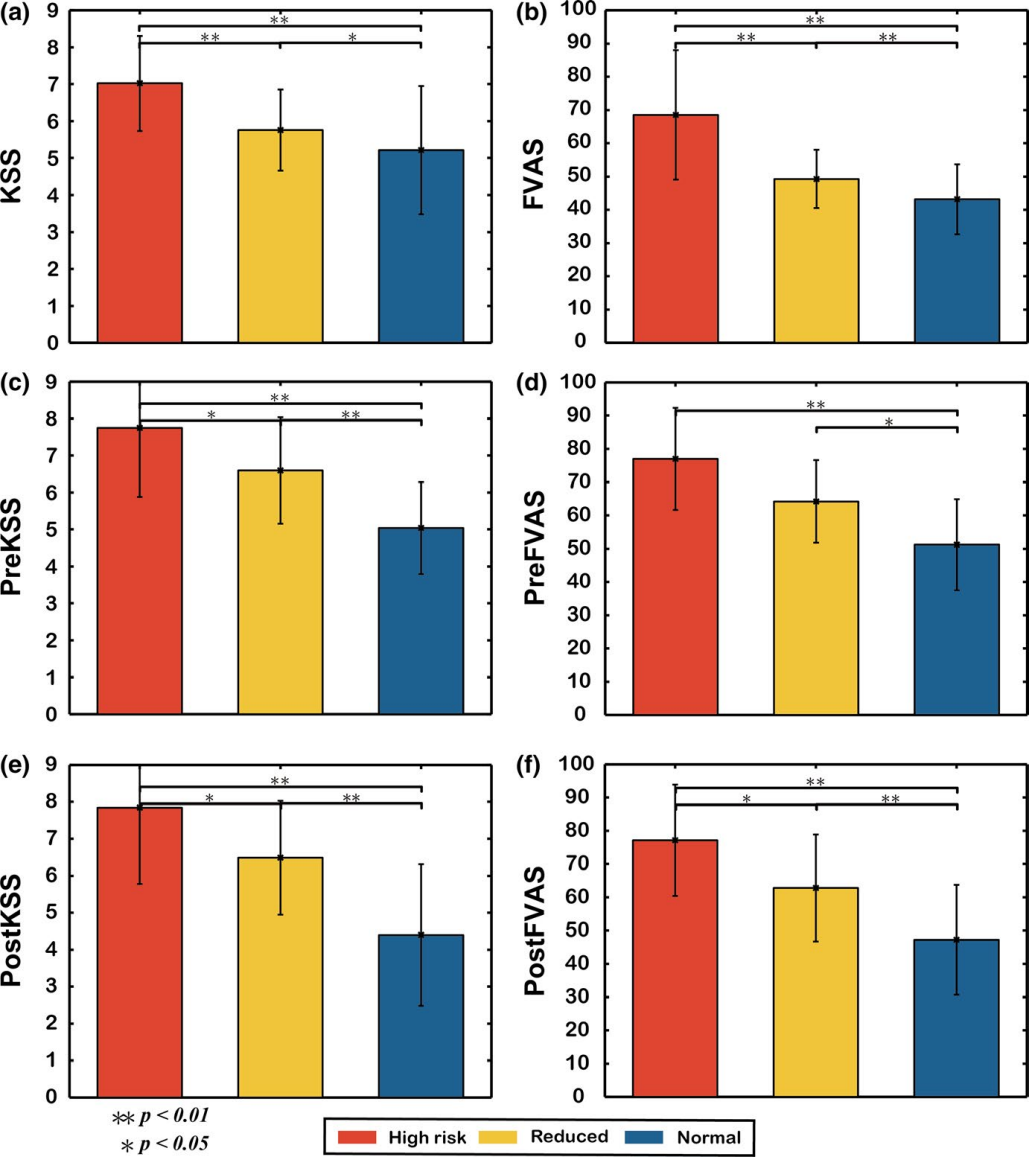
Comparison of averaged values of subjective questionnaire (KSS & FVAS) scores among three different fatigue level groups (high‐risk, reduced, and normal groups) at three time points in the day (morning, before the experiment, and after the experiment). Standard deviations are also shown. *p‐value <.05, **p‐value <.01

In Figure 3, the three fatigue groups including the high‐risk (red bars), reduced (yellow bars), and normal (blue bars) groups are compared in terms of KSS, FVAS, PreKSS, PreFVAS, PostKSS, and PostFVAS. Except the values of PreFVAS between high‐risk and reduced groups, the significant differences among all three fatigue groups can be explored as shown in Figure 3 (*p* < .05). It is worth to note that the significances between high‐risk and normal groups are always small (*p* < .01). The results show that the subjective questionnaire (KSS & FVAS) scores are significantly different across the three different fatigue levels.

### 3.2 Comparison of task performance between different levels of fatigue

**Figure 4:**
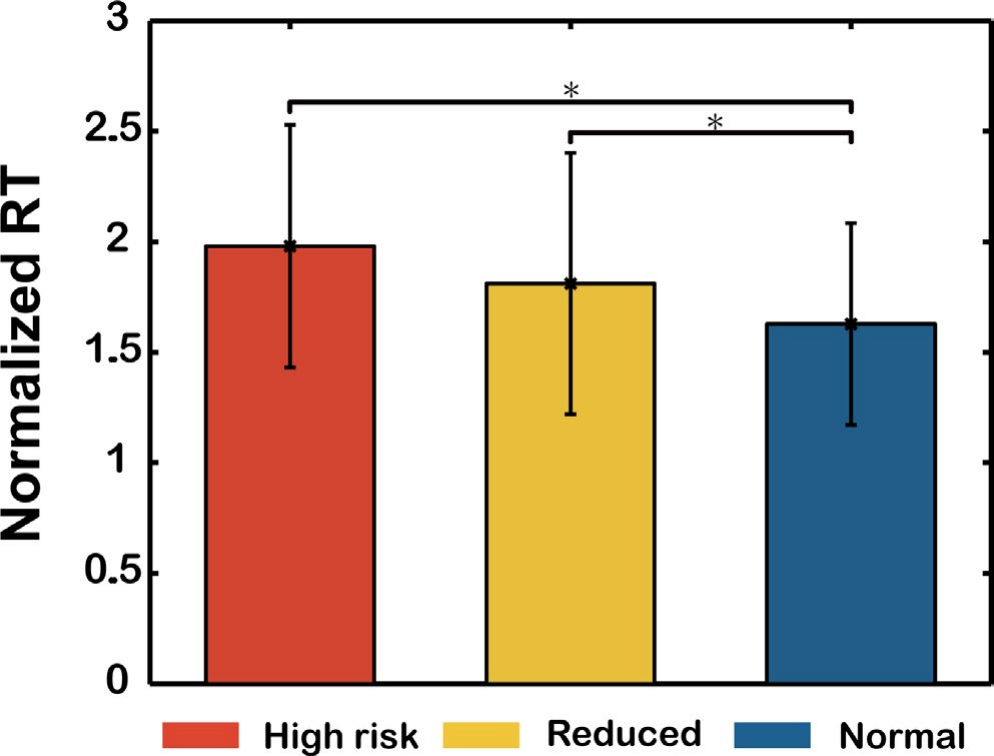
Comparison of normalized RTs of trials of lane‐keeping task among three different fatigue groups (high‐risk, reduced, and normal groups). Standard deviations are also shown. The significantly longest RTs were in the high‐risk group. *p‐value <.05

The comparisons of normalized reaction time among three fatigue groups are shown in Figure 4 (vertical axis, normalized reaction time; horizontal axis, red, high‐risk group; yellow, reduced group; blue, normal group). The reaction times were normalized by dividing the mean shortest reaction times (lowest 10% of all reaction times) of alert trials of the respective experiment. A significant difference in normalized reaction time was found between the high‐risk group and the normal group (*SE* = 0.105, *p*‐value = .014, using the Bonferroni adjustment for multiple pairwise comparisons in hierarchical linear modeling). The mean normalized reaction time significantly differed between the reduced group and the normal group, with *SE* = 0.078, *p*‐value = .015. Nevertheless, there was no significant difference in the mean normalized reaction time between the reduced group and the high‐risk group. Regarding the behavioral performance results, the normalized RTs increased with increasing fatigue levels (normal, reduced, and high‐risk).

### 3.3 Brain–behavior relationships across different levels of fatigue

**Figure 5:**
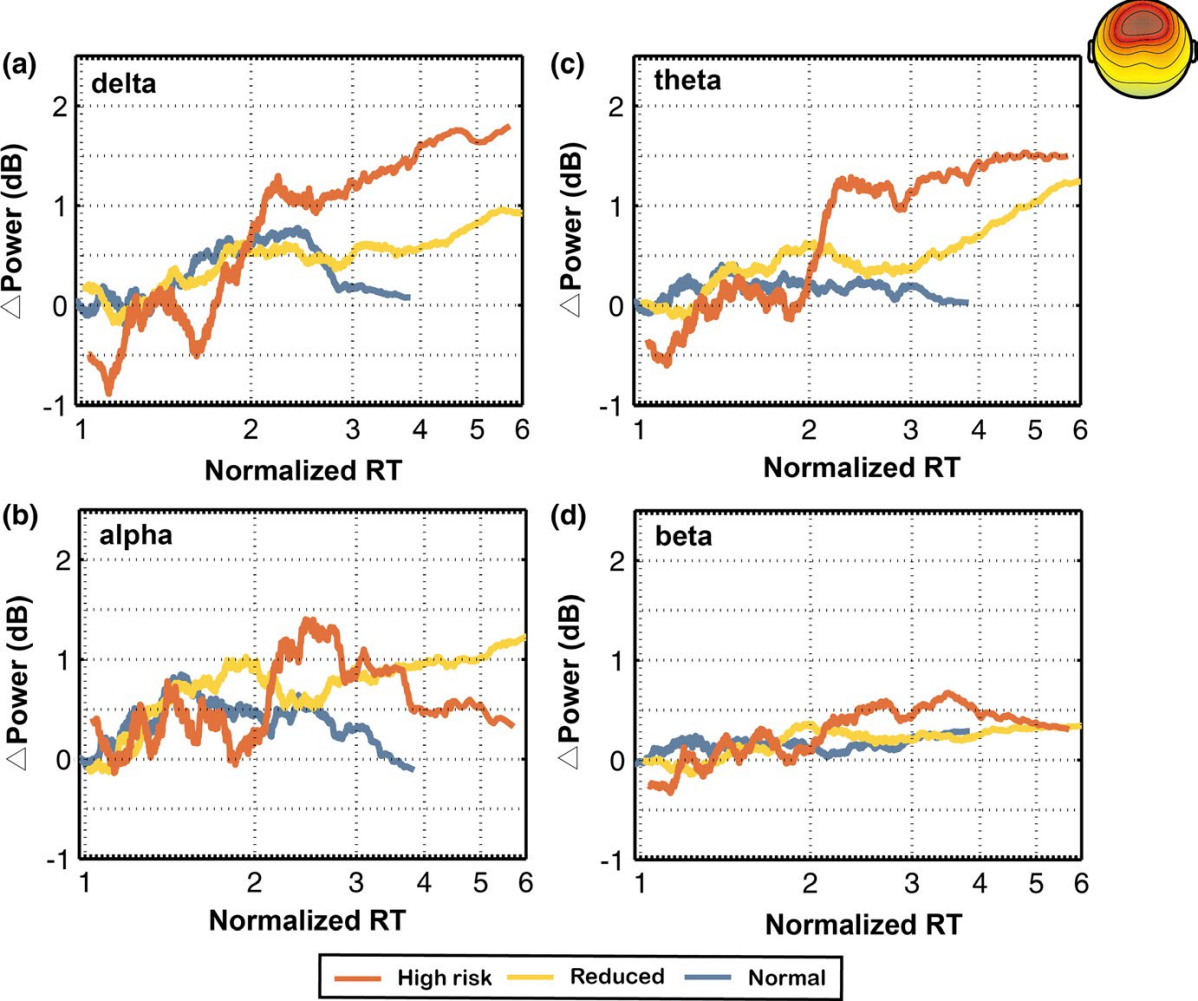
The trends of averaged component power spectra in the delta, theta, alpha, and beta bands from the frontal components among three different fatigue groups (high‐risk, reduced, and normal groups) with increasing normalized RTs. Note that the EEG power shown in this figure was calculated from the EEG data recorded in the 3 s prior to the onset of lane deviation

**Figure 6:**
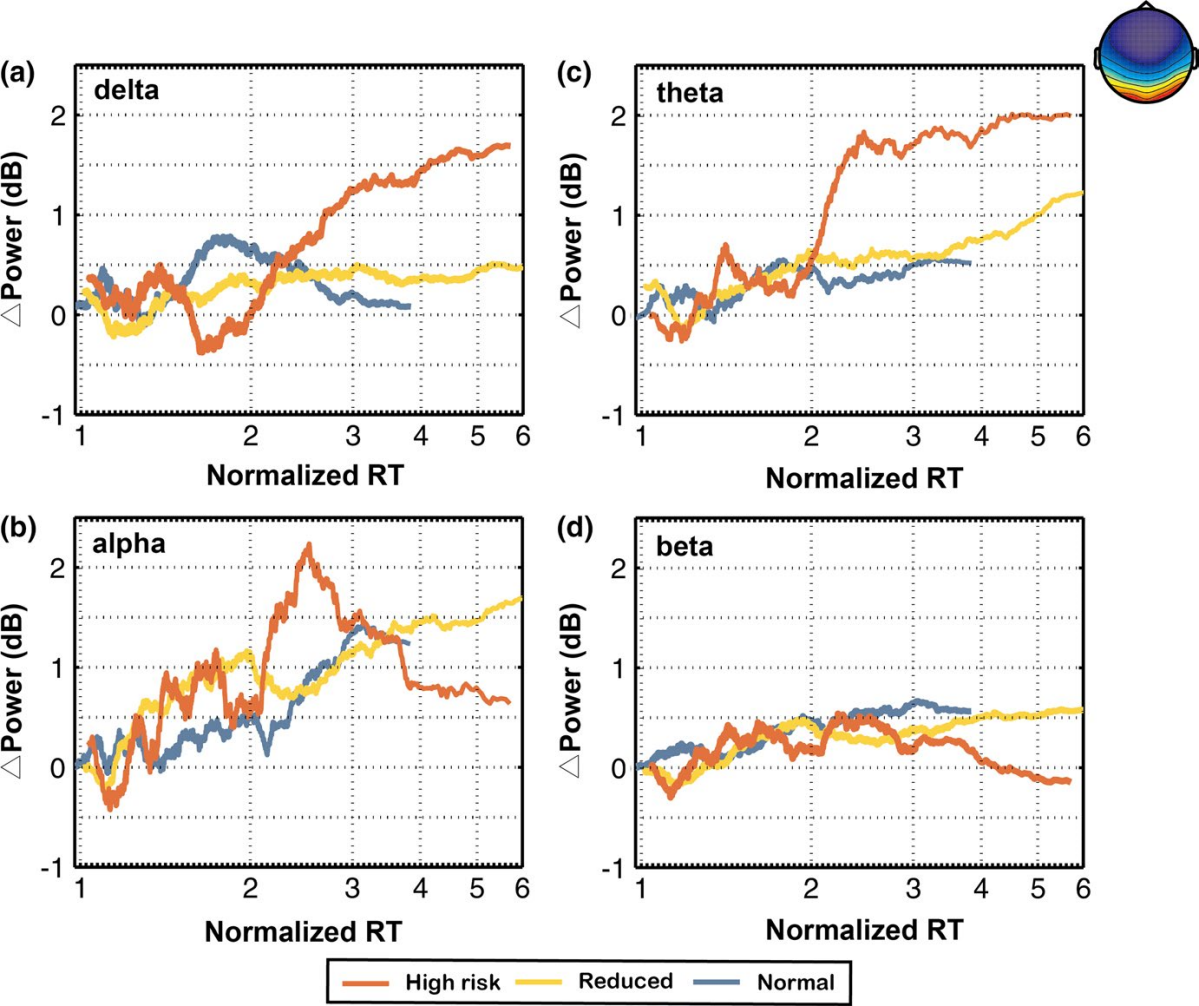
The trends of averaged component power spectra in the delta, theta, alpha, and beta bands from the occipital components among three different fatigue groups (high‐risk, reduced, and normal groups) with increasing normalized RTs. Note that the EEG power shown in this figure was calculated from the EEG data recorded over the 3 s prior to the onset of lane deviation

Figures 5 and 6 show the comparison of frontal and occipital trends among the three different fatigue groups, respectively. Figures 5a–d and 6a–d show the relation between prestimulus EEG log power in the delta, theta, alpha, and beta bands and normalized reaction time (*Y*‐axis, power in dB; *X*‐axis, RT‐sorted index and the corresponding normalized reaction time; Color: red, high‐risk state; yellow, intermediate state; blue, normal state). EEG data were collected from 17 subjects in 143 half‐hour sessions, and both measures (EEG log power and normalized reaction times) were smoothed using a window of 10% of trials.

Figure 5a shows that there was a dramatic monotonic power increase in the delta band as normalized reaction time increased in the high‐risk and intermediate group. In addition, the delta band power in the frontal regions showed an inverted U‐shaped change that was observed only in the normal group. Figure 5b shows that there was a monotonic power increase in the alpha band as normalized reaction time increased in the intermediate group. The alpha band power of the frontal regions showed an inverted U‐shaped change only in the high‐risk group. As shown in Figure 5c, the theta band power in the high‐risk group dramatically increased with normalized reaction time. The theta band power in the intermediate group increased with normalized reaction time.

Figure 6a shows that there was a dramatic monotonic increase in delta band as normalized reaction time increased in the high‐risk group. Figure 6b shows that there was a monotonic power increase in the alpha band when normalized reaction time increased in the normal and intermediate group. In addition, the alpha band power of the parietal regions showed an inverted U‐shaped change only in the high‐risk group. In Figure 6c, the theta band power in the high‐risk group dramatically increased with normalized reaction time. The theta band power in the intermediate group increased with normalized reaction time.

**Figure 7:**
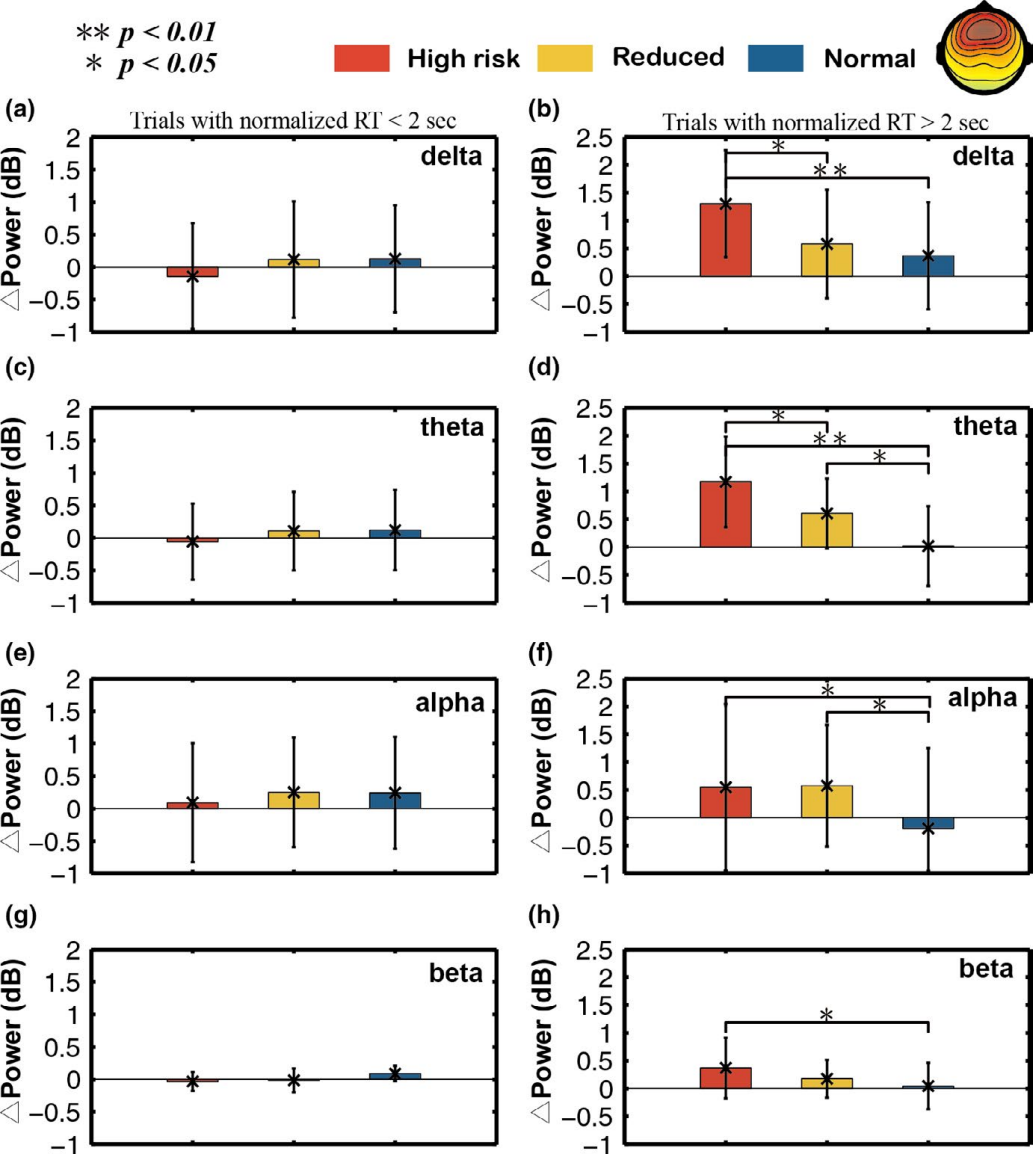
Comparison of the △power in the delta, theta, alpha, and beta bands from the frontal component among three different fatigue groups (normal, reduced, and high‐risk groups). Standard deviations are also shown. The Wilcoxon rank‐sum test was applied to determine significant differences. *p‐value <.05, **p‐value <.01

**Figure 8:**
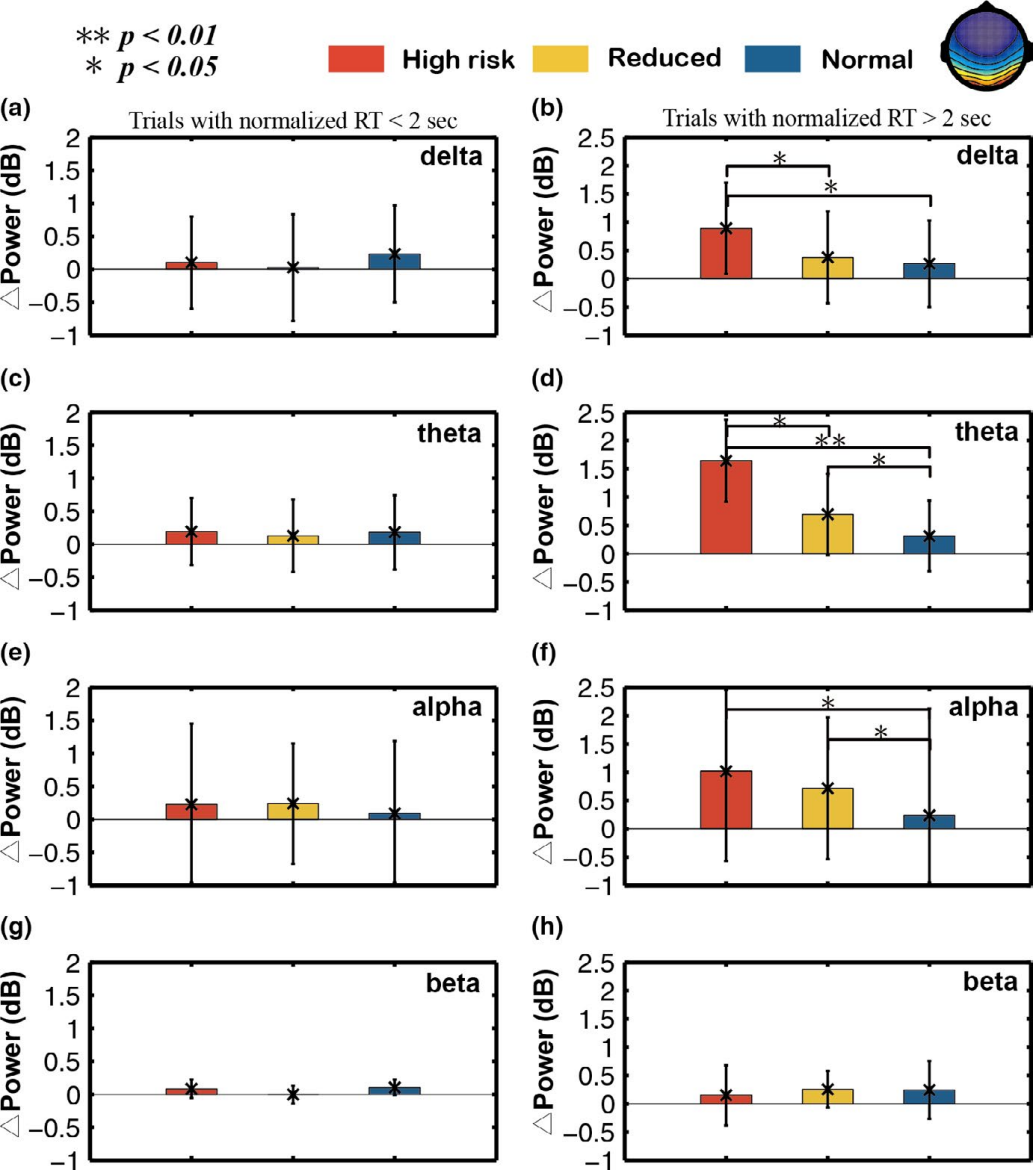
Comparison of the △power in the delta, theta, alpha, and beta bands from the occipital component among three different fatigue groups (normal, reduced, and high‐risk groups). Standard deviations are also shown. The Wilcoxon rank‐sum test was applied to determine significant differences. *p‐value <.05, **p‐value <.01

Figures 7 and 8 show the comparisons of different bands power elevations relative the baseline across three different fatigue levels group in frontal and occipital area, respectively. In each band, all trials were divided into two segments based on RT (RT <2‐s and >2‐s).

In Figure 7, the power of all bands (delta, theta, alpha, and beta) in different levels (high, reduced, and normal risks) is not significantly different among three fatigue level groups in section trials with RT <2‐s. In section trials with RT >2‐s, the power increase (*p* < .05) in high‐risk group was significantly different from reduced and normal groups, especially in theta and delta bands. Additionally, the alpha and theta power in reduced group was significantly higher (*p* < .05) than those in normal group.

In Figure 8, the power of all bands (delta, theta, alpha, and beta) in different levels (high, reduced, and normal risks) is also not significantly different among three fatigue level groups in section trials with RT <2‐s. In section trials with RT >2‐s, the power increase (*p* < .05) in high‐risk group was significantly different in delta, theta, and alpha bands from reduced and normal groups. Additionally, the alpha and theta power in reduced group were also significantly higher (*p* < .05) than those in normal group.

## 4 DISCUSSION

This study compares the power spectra between groups with different levels of fatigue to identify informative EEG features that can reflect different subjects' cognitive states. In the experiments, each subject conducted a sustained attention task (cruising on the highway) at different fatigue levels, as characterized by EEG signals, subjective questionnaires (KSS and FVAS), and objective sleep measurements (ES), to clarify the effect of real‐world fatigue on simulated driving.

### 4.1 Effect of fatigue on psychometric responses and task performance

Figure 2 shows the comparisons between daily subjective questionnaires and objective sleep measurements. The ES describes how cognitive effectiveness, reaction time, and fatigue risk are affected by sleep quality, sleep quantity, and sleep/wake timing. The relations between subjective questionnaires and objective sleep measurements across days can be observed in these experimental results. Both KSS and FVAS scores were significantly correlated with ES. The relation between KSS and ES was found to be a negative correlation, with KSS decreasing 0.03 units per unit of ES (0–100 scale). A similar pattern could be found in FVAS, which decreased 0.12 units per unit of ES. The findings lead us to believe that ES can be a reliable and objective index of fatigue levels to classify different fatigue states.

Figure 3 shows that the mean values of KSS, FVAS, PreKSS, PreFVAS, PostKSS, and PostFVAS across sessions in the high‐risk group were significantly higher than those in the normal group. The difference in questionnaires between the high‐risk and normal groups is obvious. However, the mean values of KSS, FVAS, PreKSS, PreFVAS, PostKSS, and PostFVAS across sessions in the reduced group were also different from those in the high‐risk and normal groups.

This study further compared behavioral performance (RT) across different levels of fatigue. We hypothesize that poor behavioral performance may appear at higher fatigue levels (high‐risk group). As the results show in Figure 4, the highest normalized reaction time is in the high‐risk group because the performance of subjects in the high‐risk group was influenced easily by factors such as activity, rest, and sleep.

In this study, we divided fatigue levels into three different groups by ES. The experimental results show that the high‐risk group had higher sleepiness and fatigue scores than any other group, as reported on subjective questionnaires (KSS & FVAS; Lin et al., 2018). With respect to driving behavior, we also found that subjects in the high‐risk group had the longest latency to respond to the deviation stimuli during the driving tasks as shown in Figure 4. Therefore, we found that there was a negative correlation between ES and fatigue level.

### 4.2 Effect of fatigue on brain–behavior relationships

According to the above results, in the reduced and normal groups, we found that theta and alpha band power increased, especially in occipital and frontal regions, as behavioral performance (RT) deteriorated. The theta band power in the occipital area increased significantly in the high‐risk group compared with the reduced and normal groups as behavioral performance deteriorated. In addition, an inverted U‐shaped relationship was observed in the alpha band.

Previous literature (Huang et al., 2012, 2016; Jung et al., 1997; Lin et al., 2010) indicates that theta band power increases with longer RTs during simulated driving. This upward tendency was not very clear in the normal group, whose RTs were also shorter than those of the high‐risk group because subjects in the normal group were not likely to feel drowsy. In the high‐risk group, however, we confirmed that theta band power in the occipital region clearly increases with RTs. This result not only agrees with the findings in previous studies (Huang et al., 2012, 2016; Lin et al., 2010) but also reveals that there are different brain–behavior relationships in different fatigue groups. In addition, alpha band power in the occipital region has had mixed results in previous studies. Most of the literatures (Huang et al., 2012, 2016; Lin et al., 2010) indicate that alpha band power in the occipital region increases with longer RTs, but there are other publications (Glass & Riding, 1999; Ota et al., 1996) reporting that alpha band power in occipital region has an inverted U‐shaped relationship with RTs. What we know is there have been different alpha band power results in different studies and experiments. In our research, we found that alpha band power increased with longer RTs in the normal and reduced groups. However, in the high‐risk group, we found a U‐shaped relationship between RTs and alpha band power, which indicates a sleep onset point according to previous research. From our video data, we found that the subjects in the high‐risk group usually fell asleep, which means that they entered stage 1 sleep when RTs reached a certain length. Many previous studies obtained different results in different experiments. In the current research, we further divided subjects' fatigue states into three different levels and explored the brain–behavior relationships across all three. Therefore, our study can explain the contradiction among previous studies in terms of different fatigue levels. Different fatigue states would cause different brain–behavior relationships in the real world, instead of well‐controlled settings.

Through the experimental results, this study illustrated that the brain–behavioral relationships varied depending on the levels of fatigue. In the high‐risk group (high‐fatigue level), theta band power was also a suitable feature for fatigue detection, rising as RT deteriorated in occipital and frontal regions. Hence, theta band power should be suitable for assessing drivers' vigilance levels under high‐risk conditions. Furthermore, in the reduced and normal groups (medium and low fatigue levels), the alpha band power fluctuations in the occipital area were more sensitive than the theta band power fluctuations and may be an even better feature for detecting fatigue. In addition, it is important to note that this study is different from previous studies in that it takes different fatigue levels into account. Thus, the present study explains the conflicting results of previous studies and can explore more precise brain dynamic features to predict subjects' fatigue states and behavioral performance.

## 5 CONCLUSION

This study recorded daily measurements of participants' naturally occurring sleep timing, duration, and quality, as well as their subjective perceptions of fatigue/sleepiness, and interpreted real‐world fatigue during simulated driving. The study identified the informative EEG features that reflect different fatigue levels. Furthermore, it established brain–behavior models that take fatigue into account; such models could be applied to help prevent drowsy driving.

This study also shows that the EEG spectra of trials were significantly different among the three different levels of fatigue and identifies the proper EEG features in specific brain regions for general fatigue detection. Such findings might lead to practical applications in an adaptive fatigue detection system for effectively and accurately assessing the cognitive state of human operators in daily life.

## CONFLICT OF INTEREST

None declared.

## Data Availability

The data that support the findings of this study are available from the corresponding author upon reasonable request.
